# Discoidin Domain Receptors Promote α1β1- and α2β1-Integrin Mediated Cell Adhesion to Collagen by Enhancing Integrin Activation

**DOI:** 10.1371/journal.pone.0052209

**Published:** 2012-12-20

**Authors:** Huifang Xu, Dominique Bihan, Francis Chang, Paul H. Huang, Richard W. Farndale, Birgit Leitinger

**Affiliations:** 1 National Heart and Lung Institute, Imperial College London, London, United Kingdom; 2 Department of Biochemistry, University of Cambridge, Cambridge, United Kingdom; 3 Division of Cancer Biology, Institute of Cancer Research, London, United Kingdom; University of Bergen, Norway

## Abstract

The discoidin domain receptors, DDR1 and DDR2, are receptor tyrosine kinases that bind to and are activated by collagens. Similar to collagen-binding β1 integrins, the DDRs bind to specific motifs within the collagen triple helix. However, these two types of collagen receptors recognize distinct collagen sequences. While GVMGFO (O is hydroxyproline) functions as a major DDR binding motif in fibrillar collagens, integrins bind to sequences containing Gxx’GEx”. The DDRs are thought to regulate cell adhesion, but their roles have hitherto only been studied indirectly. In this study we used synthetic triple-helical collagen-derived peptides that incorporate either the DDR-selective GVMGFO motif or integrin-selective motifs, such as GxOGER and GLOGEN, in order to selectively target either type of receptor and resolve their contributions to cell adhesion. Our data using HEK293 cells show that while cell adhesion to collagen I was completely inhibited by anti-integrin blocking antibodies, the DDRs could mediate cell attachment to the GVMGFO motif in an integrin-independent manner. Cell binding to GVMGFO was independent of DDR receptor signalling and occurred with limited cell spreading, indicating that the DDRs do not mediate firm adhesion. However, blocking the interaction of DDR-expressing cells with collagen I via the GVMGFO site diminished cell adhesion, suggesting that the DDRs positively modulate integrin-mediated cell adhesion. Indeed, overexpression of the DDRs or activation of the DDRs by the GVMGFO ligand promoted α1β1 and α2β1 integrin-mediated cell adhesion to medium- and low-affinity integrin ligands without regulating the cell surface expression levels of α1β1 or α2β1. Our data thus demonstrate an adhesion-promoting role of the DDRs, whereby overexpression and/or activation of the DDRs leads to enhanced integrin-mediated cell adhesion as a result of higher integrin activation state.

## Introduction

The extracellular matrix (ECM) physically supports cells in multicellular organisms and also signals to these cells through cell surface receptors. The interactions between ECM proteins and cell surface receptors activate a variety of signalling pathways that regulate cell behaviour and determine physiological functions. Collagens are the most abundant ECM components [1]. Collagen molecules are composed of three α chains characterized by repetitive G-X-X’ sequences, wherein the X position is often occupied by proline and X’ by 4-hydroxyproline (O). The three α chains coil around each other to form a right-handed triple-helical structure. In tissues, fibrillar collagens such as collagens I, II or III further assemble into fibrils and fibres, providing mechanical support. In addition to this main architectural role, collagens play a regulatory role in many cellular processes, such as cell adhesion, migration, growth and wound healing, which is achieved by interacting with collagen receptors that recognize specific motifs within the collagen triple helix [2]. Two major types of collagen-binding receptors are widely distributed in mammalian tissues: collagen-binding β1 integrin family members and the discoidin domain receptors (DDRs).

Integrins are a major class of ECM receptors for cell adhesion [3]. They are heterodimeric transmembrane glycoproteins that are composed of non-covalently associated α and β subunits. Collagen-binding integrins belong to the β1 integrin subfamily. There are four collagen-binding integrins in mammalian cells: α1β1, α2β1, α10β1 and α11β1 [2], [4], [5]. The α subunits of these collagen receptors have an inserted (I) domain in their extracellular region that contains the collagen-binding site. Collagen-binding integrins recognize specific amino acid motifs within the collagen triple helix. Utilisation of synthetic triple-helical peptides and comprehensive screening using libraries of overlapping collagen-like peptide “Toolkits” has enabled the identification of a number of integrin binding motifs within fibrillar collagens [6]. GFOGER was the first identified high-affinity binding motif for both α1β1 and α2β1 integrins [7], [8], [9]. Later on, a series of GxOGER motifs were identified as α2β1 integrin binding motifs, with the identity of x determining the affinity for integrins [10], [11]. Within GxOGER motifs, the affinity for α2β1 decreases in order for x = F>L>M>A. We recently also reported several α1β1 integrin-specific motifs, with the GLOGEN sequence being the most potent ligand for this receptor [12].

The discoidin domain receptors, DDR1 and DDR2, are a subfamily of receptor tyrosine kinases. Both DDR1 and DDR2 are single-span transmembrane proteins, with an extracellular region containing an N-terminal discoidin homology (DS) domain, which contains the collagen binding site, and a second globular domain, the DS-like domain with structural similarity to the DS domain [13], [14], [15]. The cytoplasmic domain contains a large juxtamembrane domain and a C-terminal tyrosine kinase domain. Both DDRs bind a number of collagens with distinct specificity [16], [17], [18], [19]. The DDRs exist as pre-formed homodimers independent of ligand binding [20], [21]. Collagen binding induces receptor autophosphorylation with distinctly slow and prolonged kinetics [16], [17]. Both DDRs bind to the motif GVMGFO, present in collagens I–III [22], [23]. Synthetic triple-helical collagen-like peptides incorporating GVMGFO can effectively induce DDR autophosphorylation with the same kinetics as full-length collagen [22], [23]. DDRs and integrins employ fundamentally different collagen binding modes [14], [24], the former being cation-independent, and recognize distinct binding motifs in the fibrillar collagens.

The DDRs are widely expressed in mammalian tissues and play important roles in development. For example, DDR1 is essential for mammary gland development [25], while DDR2 regulates the growth of long bones [26]. Mutations in *DDR2* cause a rare form of severe growth retardation [27], [28]. The DDRs are also involved in disease progression in a wide variety of diseases, including fibrotic disorders, atherosclerosis, arthritis and many types of cancer [29], [30]. While integrins are the primary mediators of cell adhesion and migration, previous work has shown that DDR1 can influence these processes in a cell type-dependent manner. For example, DDR1 overexpression promoted cell adhesion to collagen in several cell types such as leukocytes, glioma cells and pituitary adenoma cells [31], [32], [33], while the absence of DDR1 reduced adhesion to collagen in smooth muscle cells and mesangial cells [34], [35]. In almost all cellular systems described so far, DDR1 has been reported as pro-adhesive. However, in MDCK cells, DDR1 was shown to inhibit cell adhesion and migration [36]. DDR1 was further found to inhibit cell spreading in several cell types [37], [38].

Little is known about the interplay between the DDRs and collagen-binding integrins, but cross talk between them has been observed in some cellular systems. While DDR1 activation by collagen occurs independently of β1 integrins [39], some of the DDR1 downstream signalling pathways have been shown to converge with integrin-induced pathways. For example, in MDCK cells, DDR1 binding to collagen negatively regulates the function of α2β1, involving the phosphatase SHP-2 and suppression of the activation of transcriptional activators Stat1 and Stat3 [36]. DDR1 signalling was also found to inhibit integrin-initiated cell spreading in these cells by blocking Cdc42 activity [37]. In pancreatic cancer cells, on the other hand, cooperation of both DDR1 and α2β1 is required to mediate collagen-induced epithelial-to-mesenchymal transition, to transmit a signal to JNK, which in turn up-regulates N-cadherin expression and promotes cell scattering [40]. A more recent study also showed that DDR1 and α2β1 cooperate to promote the self renewal of mouse embryonic stem cells through cell cycle regulation [41].

As described above, DDR1 has been shown to be involved in cell adhesion to collagen in some cell types, but the contribution of DDR2 to this process is not clear. Furthermore, previous studies have not addressed whether the DDRs can function as true adhesion receptors or exert their function indirectly through influencing integrin-mediated cell adhesion. Since integrins and DDRs both bind to collagen, the contribution of each receptor type to collagen-initiated cellular events is difficult to establish. Here we have made use of receptor-selective synthetic triple-helical collagen-like peptides, to address the individual contributions of the two collagen receptor types to cell adhesion. To this end, we have used HEK293 cells that stably express DDR1 or DDR2. Our data show that, while β1 integrins predominate in the cell adhesion to collagen I, the DDRs also contribute, through specific DDR-binding sites within collagen. This activity is independent of DDR receptor signalling and occurs with limited cell spreading. Furthermore, we observed DDR-induced positive modulation of α1β1 and α2β1 integrin-mediated cell adhesion to medium- and low-affinity integrin ligands without regulating the cell surface expression levels of the integrin subunits. These results provide evidence that DDR signalling can regulate the affinity of β1 integrins.

## Materials and Methods

### Cell Culture and Cell Lines

Human embryonic kidney (HEK) 293 cells (ATCC, Manassas, VA) and stably transfected HEK293 cells were cultured in Dulbecco’s modified Eagle’s medium/F12 nutrient mixture (Invitrogen) supplemented with 2 mM L-glutamine and 10% fetal bovine serum at 37°C, 5% CO_2_.

### Chemicals and Reagents

Bovine serum albumin was obtained from Fisher Scientific Ltd (Loughborough, UK). Collagen I (acid-soluble from rat tail; C-7661) was purchased from Sigma (Gillingham, UK). Calcein-AM was purchased from Invitrogen (Paisley, UK). The antibodies (Abs) and their sources were as follows: rabbit-anti-DDR1 (SC-532) from Santa Cruz Biotechnology Inc. (Santa Cruz, CA); goat-anti-DDR2 (AF2538) from R&D Systems (Abingdon, UK); mouse anti-phosphotyrosine, clone 4G10, from Upstate Biotechnology (Lake Placid, NY); mouse anti-integrin alpha 1, clone FB12, from Millipore Chemicon (Southampton, UK); mouse anti-integrin alpha 2, clone AK7, from AbD Serotec (Oxford, UK). Mouse anti-integrin beta 1, clone P5D2, was purified from hybridoma cells obtained from the Developmental Studies Hybridoma Bank, University of Iowa. Mouse anti-DDR1 Abs, were generated in our lab [15]. Secondary Abs were as follows: rabbit-anti-goat Ig horseradish peroxidase conjugated (Zymed Laboratories Inc., San Francisco, CA); goat-anti-rabbit Ig horseradish peroxidase conjugated (P0448, DAKO A/S, Denmark); sheep-anti-mouse Ig-horseradish peroxidase (Amersham Biosciences UK, Chalfont St Giles, UK) and goat anti-Mouse IgG FITC-conjugated (F-9006, Sigma, Gillingham, UK).

### Synthetic Triple-helical Collagen-derived Peptides

The sequences of the peptides used in this study are shown in Table S1. In the DDR-specific peptide, methionine was replaced by the more stable isosteric analogue, Norleucine, because this increased the apparent affinity to DDR2 [14]. Peptides were synthesized by Fmoc (N-(9-fluorenyl)methoxycarbonyl) chemistry as C-terminal amides on TentaGel R RAM resin in an Applied Biosystems Pioneer automated synthesizer and purified as described [10]. All peptides were verified by mass spectrometry and shown to adopt triple-helical conformation by polarimetry.

### Generation of Stable DDR1b and DDR2 Expressing HEK293 Cells

HEK293 cells were transfected with cDNAs encoding DDR1b or DDR2, in pcDNA3.1/zeo (Invitrogen) by the calcium phosphate precipitation method [42]. 48 h after transfection, cells were selected in 400 µg/ml zeocin. Resistant cells were pooled and analyzed for DDR expression by Western blotting.

### Cell Adhesion Assay

All adhesion assays were carried out in 96-well Immulon 2HB plates (Fisher Scientific). The plates were coated either with collagen I or with collagen-derived peptides, both at 10 µg/ml, overnight at 4°C. The wells were then washed twice with PBS and blocked using 1% (w/v) BSA in PBS for 2 h at room temperature followed by two washes with PBS. Cells were incubated with 5 µM Calcein-AM in serum-free medium containing 0.1% BSA, for 30 min at 37°C. Cells were then seeded onto substrate-coated 96-well plates (5×10^5^ cells/well, in serum-free medium containing 0.1% BSA) after removing unbound calcein-AM by two washes. In the inhibition experiments, calcein-stained cells were pre-incubated with Abs for 30 min at 37°C prior to seeding onto 96-well plates. Cells were normally allowed to adhere for 1 h unless stated otherwise. Non-adherent cells were removed by four washes with HEPES buffer (10 mM HEPES pH 7.4, 140 mM NaCl, 4.7 mM KCl, 0.65 mM MgSO_4_, 1.2 mM CaCl_2_), and the remaining cell-bound fluorescence was measured on a FLUOStar Galaxy plate reader (BMG Lab Technologies) at 485 nm excitation and 520 nm emission. Cell adhesion was calculated as percentage with respect to the seeding cell fluorescence (set to 100% for each cell line).

### Interference Reflection Confocal Microscopy

Collagen I at 2 µg/ml or collagen-derived peptide at 10 µg/ml was coated onto 35 mm glass-based ibidi µ-dishes (IB-81158, Thistle Scientific, Glasgow UK) overnight at 4°C. The dishes were then blocked with 1% BSA in PBS for 1 h at room temperature followed by two washes in PBS. DDR-expressing HEK293 cells were seeded and allowed to adhere for 1 h at 37°C. After three washes with HEPES buffer as above, attached cells were fixed in 4% PFA for 15 min at room temperature followed by two washes in PBS. The attachment sites between the cell membrane and the substrates were viewed under a Zeiss LSM-510 inverted confocal microscope using interference reflection microscopy (IRM) settings. Images were taken and analyzed using Zeiss LSM510 software and image browser. For quantification of cellular contact areas with the substratum HCImage software (Hamamatsu Corp, Bridgewater, NJ) was used. The cell boundary and contact area was identified and measured using built-in tools.

### Flow Cytometry

Cells were grown in 6-well tissue culture plates for 24 h before being dissociated with non-enzymatic cell dissociation solution (Sigma) and resuspended in PBS containing 1% BSA. The cells were incubated for 30 min on ice with primary monoclonal antibodies (mAbs) at 10 µg/ml in 100 µl PBS/BSA. Cells were then washed three times with PBS/BSA and incubated with FITC-conjugated goat anti-mouse IgG (F-9006, Sigma) for 30 min on ice. After three washes as above, the cells were resuspended in 2% formaldehyde in PBS. Data were subsequently collected on a BD LSRFortessa cell analyzer using BD FACSDiva software 6.0 (Becton, Dickinson and Company), and further analyzed on FlowJo software 7.6.4 (Tree Star, Inc. USA).

### DDR Autophosphorylation Assay

The assay was performed as described before [42]. Briefly, DDR-expressing HEK293 cells or empty-vector control HEK293 cells were grown in 12-well plates. 24 h later, the cells were incubated with serum-free medium for 16 h. Cells were then stimulated with collagen I (at 10 µg/ml) or different collagen peptides (at 100 µg/ml) for 90 min, at 37°C. In the inhibition experiment, cells were pre-incubated with dasatinib for 30 min at 37°C prior to collagen stimulation. Cells were lysed in 1% Nonidet P-40, 150 mM NaCl, 50 mM Tris, pH 7.4, 1 mM EDTA, 1 mM phenylmethylsulfonyl fluoride, 50 µg/ml aprotinin, 1 mM sodium orthovanadate, 5 mM NaF. Aliquots of the lysates were analyzed by SDS-PAGE followed by blotting onto nitrocellulose membranes. The duplicate blots were probed with either anti-phosphotyrosine mAb or anti-DDR Abs followed by corresponding secondary Abs. Detection was performed using Enhanced Chemiluminescence Plus (Amersham Biosciences) on an Ettan DIGE Imager (GE Healthcare Biosciences).

### Western Blotting

Cells grown in 12-well tissue culture plates were lysed as above. Aliquots of cell lysates were loaded onto SDS-PAGE gels followed by blotting onto nitrocellulose membranes. The duplicate blots were probed with anti-DDR1, anti-DDR2 or anti-tubulin followed by corresponding horseradish peroxidase-conjugated secondary Abs. Detection was carried out using Enhanced Chemiluminescence Plus reagent (Amersham Biosciences) on an Ettan DIGE Imager (GE Healthcare Biosciences).

## Results

### DDRs Mediate Cell Attachment to a DDR-specific Collagen-derived Triple-helical Peptide

To investigate the roles of the DDRs in cell adhesion to collagen, we used HEK293 cells - cells that generally display relative weak adhesion to various substrates, in particular to collagen I (e.g. ref [43]). To this end, HEK293 cells were stably transfected with full length DDR1b or DDR2 cDNA. Overexpression of the two receptors was verified by Western blotting and flow cytometry analysis (Fig. S1 A, B). As expected from our previous results in transiently transfected HEK293 cells [22], [23], DDR receptors expressed in these stable cell lines could be activated by either collagen I or the DDR-specific collagen peptide ligand GPRGQOGVNleGFO, but not by the integrin-specific collagen peptide GFOGER (Fig. S1 C, D).

**Figure 1:**
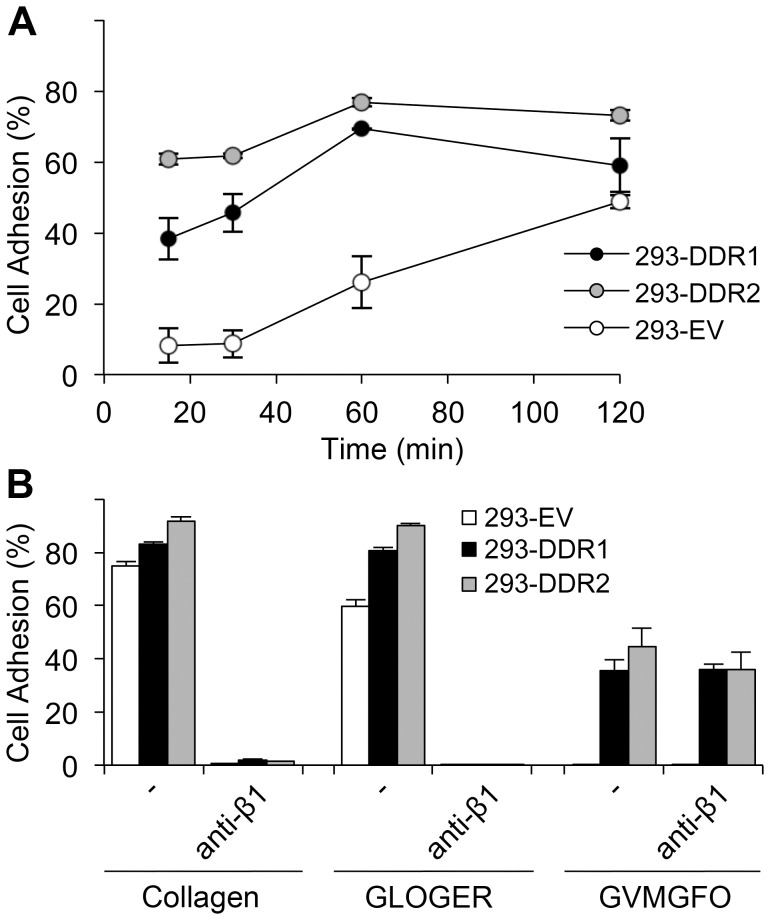
Cell adhesion of DDR-overexpressing HEK293 cells to collagen I or collagen-derived peptides. (A) Time course of cell adhesion to collagen I. Cell adhesion assays were carried out in 96-well plates with fluorescently labeled cells. 293-DDR cells or empty-vector control cells (293-EV) were allowed to adhere to collagen I-coated wells for the indicated duration at 37°C. Cell adhesion was measured and calculated as described in Materials and Methods. Data shown are representative of four independent experiments, each performed in triplicate. (B) Cell adhesion to collagen I, integrin-specific peptide (GLOGER) or DDR-specific peptide GPRGQOGVNleGFO (GVMGFO). Cells were allowed to adhere to different substrates for 1 h at 37°C in the presence or absence of 10 µg/ml β1-integrin blocking mAb P5D2. The data are representative of at least four independent experiments, each performed in triplicate. The error bars indicate the sample standard deviation.

We noticed that DDR-overexpressing cells had a tendency for increased cell adhesion to collagen I, compared with non-transfected HEK293 and empty-vector control cells. This was most noticeable at early time points of adhesion (Fig. 1A). While DDR-expressing cells showed a robust and reproducible response to collagen I, empty-vector control cells showed more variability in terms of percentage of cell adhesion at 1 h incubation, with levels sometimes closer to the DDR-overexpressing cells. Cell adhesion to collagen I was completely inhibited by the use of an anti-β1 integrin blocking Ab, indicating that adhesion to collagen I is mediated by β1 integrin family members in all tested cell lines (Fig. 1B). Similarly, and as expected, cell adhesion to integrin-specific collagen ligands such as GFOGER or GLOGER was also abolished by the anti-β1 blocking Ab. Our results further showed that DDR-expressing cells, but not empty-vector control cells, exhibited moderate levels of adhesion to the DDR ligand GPRGQOGVNleGFO, and this adhesion was not affected by the anti-β1 Ab (Fig. 1B). Cell adhesion to GPRGQOGVNleGFO was more variable than adhesion to collagen I, with about 20–40% of total cells adhering to the DDR ligand. These findings indicate that although adhesion to collagen I is predominantly mediated by collagen binding β1 integrins, DDR receptors themselves can also mediate cell binding to DDR-specific binding sites in collagen.

### DDR-mediated Cell Binding to GPRGQOGVNleGFO is Independent of Receptor Signalling

**Figure 2:**
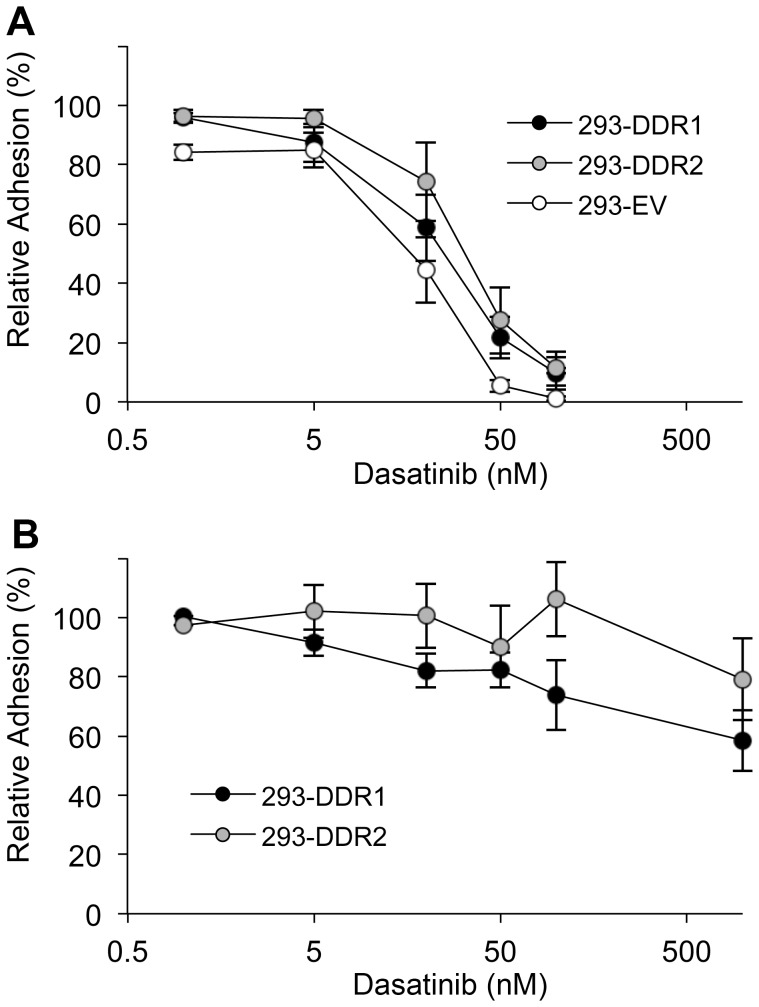
Dasatinib inhibits cell adhesion to integrin ligand GLOGER but not to DDR ligand GVMGFO. 293-DDR cells or empty-vector control cells (293-EV) were allowed to adhere to the integrin-specific peptide GLOGER (A) or to the DDR-specific peptide GPRGQOGVNleGFO (GVMGFO) (B) for 1 h at 37°C in the presence or absence of dasatinib at the indicated concentrations. The data are the means ± SEM of 3–5 independent experiments, each performed in triplicate. Due to variability of cell adhesion to GVMGFO, the data are normalised to adhesion in the absence of dasatinib, which was set to 100%.

To determine whether DDR signalling is involved in the DDR-mediated cell adhesion, we tested the effect of a receptor tyrosine kinase inhibitor, dasatinib, on cell adhesion. Dasatinib was previously reported to inhibit in vitro Src kinase activity with an IC50 of 3 nM and suppressed downstream focal adhesion kinase signalling pathways and integrin-mediated cell adhesion to fibronectin at nM concentration levels [44]. Consistent with this, our data show that adhesion to the integrin ligand GLOGER was efficiently inhibited by dasatinib at 50 nM (Fig. 2A).

The DDRs are also targets of dasatinib [45], [46]. Using cell-free kinase assays and collagen-induced receptor phosphorylation as read-outs, Day et al (2008) reported potent DDR kinase inhibition by dasatinib, with IC50s of 0.5–1.4 nM and 1.4–5 nM, respectively, for DDR1b and DDR2 [46]. Consistent with these findings, our cell-based receptor autophosphorylation assay also showed substantial inhibition of DDR autophosphorylation by dasatinib in the low nM range (Fig. S2 A, B). However, cell adhesion to the DDR ligand GPRGQOGVNleGFO remained largely unaffected by dasatinib, even at concentrations several-fold higher than required for inhibition of DDR signalling (Fig. 2B), suggesting that receptor signalling is not involved in DDR-mediated cell attachment to GPRGQOGVNleGFO.

**Figure 3:**
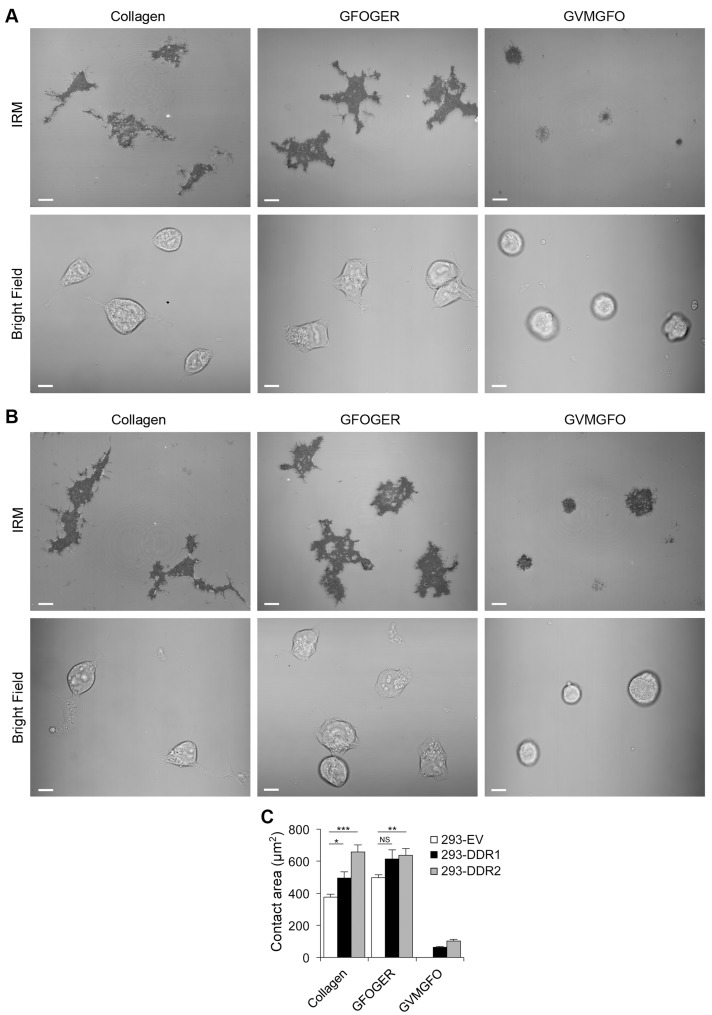
Cell spreading on collagen I, integrin ligand GFOGER or DDR ligand GVMGFO. 293-DDR1 cells (A) or 293-DDR2 cells (B) were allowed to adhere to substrate-coated ibidi dishes as described in Materials and Methods. Cell spreading was observed using interference reflection microscopy (IRM) to view the attachment sites between the cell membrane and the substrate. (A) and (B): Reflective images show the footprint of cell-substrate contact areas (upper panels). The bright field images show intact cells (lower panels). White bars, 10 µm. The data are representative of three independent experiments, in which 3–6 independent optical fields were taken. (C) Quantification of contact area showing the means ± SEM of 20–45 cells for each condition. Statistical analysis was performed using unpaired, two tailed, T-test with unequal variances, and statistical significance was defined as P<0.05 (*), P<0.01 (**), and P<0.001 (***).

A further investigation into cell spreading on these adhesion substrates, using interference reflection microscopy, revealed that all three cell lines were well spread on collagen I or the integrin ligand GFOGER, with long protrusions (images shown for DDR-overexpressing cells, in Figs 3A and 3B), whereas DDR-overexpressing cells on the GPRGQOGVNleGFO substrate were mostly rounded up, exhibiting limited cell spreading with very few short protrusions. Quantification of the cellular contact area with the substrata confirmed that DDR-expressing cells made only minimal contact to the GPRGQOGVNleGFO substrate (Fig. 3C). This analysis further revealed significant increases in contact area to collagen I by DDR-overexpressing cells compared with empty-vector control cells, indicating increased cell spreading on collagen I. On GFOGER, DDR2 expressing cells showed increased contact areas compared with empty-vector control cells.

Taken together, these data show that cell adhesion to integrin-specific ligands, as expected, involves integrin-initiated signalling events (which can be blocked by dasatinib) that lead to cell spreading. DDR-mediated cell attachment to GPRGQOGVNleGFO, on the other hand, does not involve DDR-initiated signalling and cells are unable to spread on the substrate. Thus, while DDRs can mediate cell attachment to collagen, they appear unable to mediate firm adhesion.

### Overexpression of DDRs Promotes Integrin-mediated Cell Adhesion

**Figure 4:**
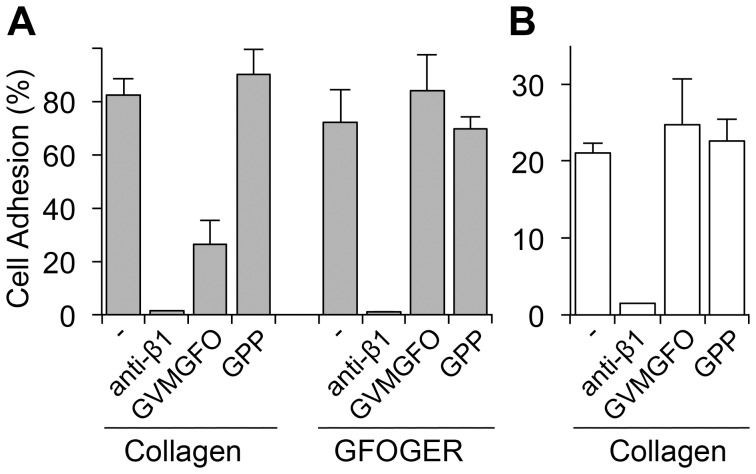
Blocking the interaction with collagen via the GVMGFO site reduces cell adhesion to collagen I in DDR-overexpressing cells. 293-DDR2 cells (A) or 293 empty-vector control cells (B) were pre-incubated with β1-integrin blocking mAb P5D2 (10 µg/ml), high affinity DDR-binding peptide GPRGQOGVNleGFO (GVMGFO at 100 µg/ml) or control peptide GPP (100 µg/ml) for 30 min before seeding onto the indicated substrates. Cell adhesion was then measured after 1 h incubation at 37°C. The data are representative of at least three independent experiments, each performed in triplicate. The error bars indicate the sample standard deviation.

Our previous study showed that GVMGFO-containing peptides could efficiently inhibit binding of recombinant DDR2 to immobilised collagen [22]. We therefore examined cell adhesion to collagen in the presence of the GPRGQOGVNleGFO peptide in solution. As shown in Fig. 4A, after pre-incubating 293-DDR2 cells with this peptide, a dramatic decrease in cell adhesion to collagen I was observed, which was not seen when cells were incubated with the control peptide GPP, an inert peptide consisting of GPP repeats that does not bind to cells. Soluble GPRGQOGVNleGFO, as expected, was unable to compete off the integrin-mediated interaction of 293-DDR2 cells with immobilised GFOGER (Fig. 4A). Soluble GPRGQOGVNleGFO further had no effect on empty-vector control cells adhering to collagen I, further confirming specificity of the blocking effect (Fig. 4B). Thus, although cell adhesion to collagen I is predominantly mediated by β1 integrins (Fig. 1B), blocking the DDR2-collagen interaction via the GVMGFO-containing site reduces integrin-mediated cell adhesion to collagen I.

**Figure 5:**
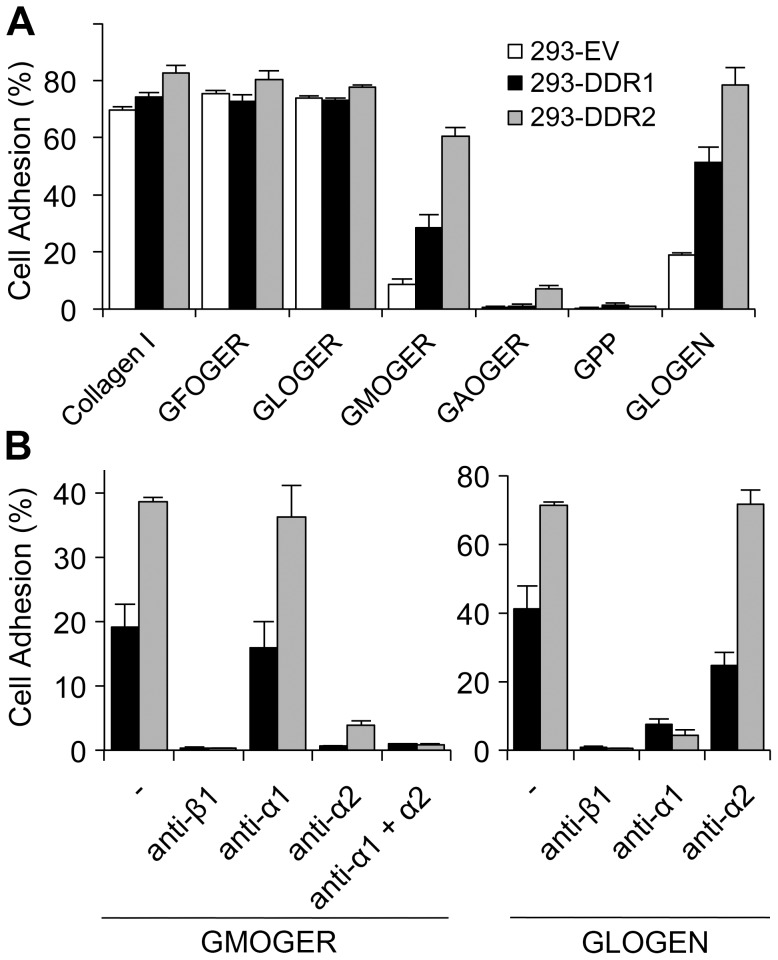
DDR-overexpressing cells exhibit increased cell adhesion to medium-affinity integrin-selective collagen peptides. (A) Cell adhesion to collagen I or a range of integrin-specific collagen peptides (GxOGERs and GLOGEN) with different integrin-binding affinities. Cells were allowed to adhere to the different substrates for 1 h at 37°C. Data shown are representative of at least four independent experiments, each performed in triplicate. The error bars indicate the sample standard deviation. (B) Blocking of cell adhesion to GMOGER or GLOGEN with anti-integrin blocking Abs. Cells were allowed to adhere to the indicated substrates in the presence or absence of 10 µg/ml indicated anti-integrin mAbs. The data are representative of three independent experiments, each performed in triplicate. The error bars indicate the sample standard deviation.

Because DDR-overexpressing cells showed a tendency for increased integrin-mediated cell adhesion to collagen, we were prompted to examine cell adhesion to a range of integrin-specific collagen-derived peptides that display different affinities for collagen-binding integrins. Our peptide set contained GxOGER motifs or GLOGEN [10], [11]. GxOGER motifs are the preferred ligands for α2β1 integrin, with their affinity for α2β1 decreasing in the order of GFOGER>GLOGER>GMOGER>GAOGER [11]. GLOGEN, on the other hand, is the most potent ligand for α1β1 integrin in the fibrillar collagen sequences, with a higher affinity for α1β1 than GFOGER [12]. Consistent with results obtained with HT1080 cells and platelets [11], our HEK293 cell lines displayed distinct adhesion profiles to the GxOGER peptide set. Adhesion of all three cell lines was maximal to the high affinity ligand GFOGER and GLOGER, with adhesion comparable to adhesion to collagen I (Fig. 5A). Adhesion to GMOGER was reduced compared with the GLOGER and GFOGER substrates, and adhesion to GAOGER was very poor. Compared with empty-vector control cells, DDR-expressing cells exhibited an obvious increase in cell adhesion to medium or low-affinity integrin ligands, in particular to GMOGER and GLOGEN (Fig. 5A). While adhesion to GFOGER was always robust and maximal (∼80% of input cells for all three cell lines), adhesion to lower affinity substrates was somewhat more variable from experiment to experiment, as would be expected since cell attachment to these substrates will be marginal in these manually-washed adhesion assays. However, in all of our experiments, adhesion decreased in the order of GFOGER>GLOGER>GMOGER>GAOGER, and DDR-expressing cells consistently showed higher adhesion to GMOGER and GLOGEN compared with empty-vector control cells.

We then carried out functional Ab blocking assays to identify the integrin α subunits responsible for mediating increased cell adhesion in the 293-DDR cells. While adhesion to collagen I could only be blocked by a combination of anti-α1 and anti-α2 Abs (Fig. S3A), adhesion to GLOGER was effectively blocked by anti-α2 but remained largely unaffected by anti-α1 (Fig. S3B). As shown in Fig. 5B, adhesion of DDR-transfected cells to GMOGER was blocked by anti-α2 but not by anti-α1. In contrast, cell adhesion to GLOGEN was inhibited by anti-α1 rather than by anti-α2 (Fig. 5B). Together, these experiments indicate that overexpression of the DDRs in HEK293 cells leads to increased cell adhesion that can be mediated by either α2β1 integrin (to GMOGER), or α1β1 integrin (to GLOGEN).

**Figure 6:**
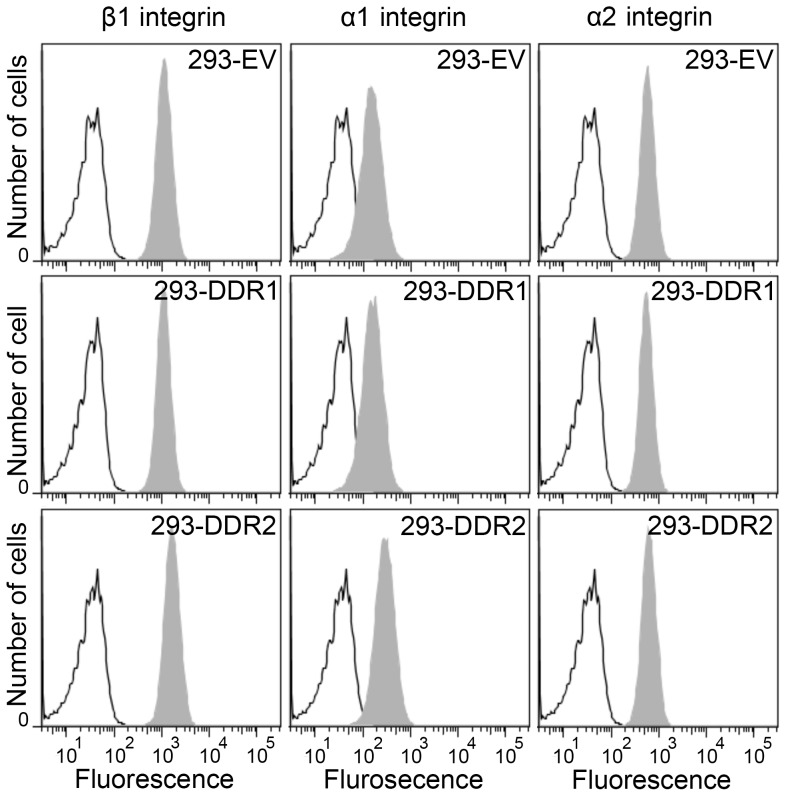
Cell surface expression of collagen-binding integrin subunits in DDR-expressing or empty-vector control HEK293 cells. The cells were stained on ice with 10 µg/ml primary mAbs followed by FITC-conjugated goat anti-mouse IgG and analysis by flow cytometry. Open black histograms, secondary Ab only; grey filled histograms, integrin subunits as indicated. Shown are representative data of three independent experiments.

To test whether the expression level of α1β1 or α2β1 was altered in the DDR-expressing cell lines, we analyzed cell surface integrin levels using flow cytometry. As shown in Fig. 6, cell surface expression levels of the α1, α2 and β1 subunits were very similar among the three studied cell lines, with perhaps a small increase in β1 and α1 subunits in 293-DDR2 cells, compared with 293-DDR1 and control cells. Thus, our data suggest that overexpression of DDRs promotes both α1β1 and α2β1 integrin-mediated cell adhesion to medium affinity integrin-specific collagen peptides without regulating α1β1 or α2β1 cell surface levels.

### Activation of DDRs Enhances α1β1 and α2β1 Integrin-mediated Cell Adhesion

**Figure 7:**
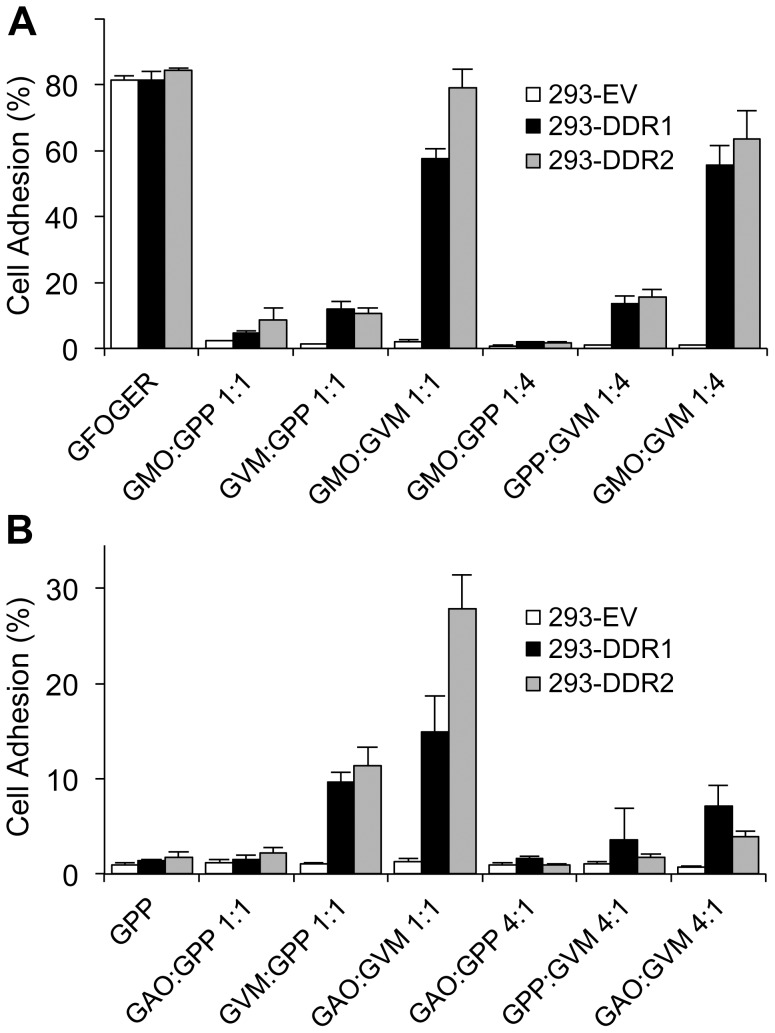
Combinations of integrin and DDR ligands support enhanced cell adhesion of 293-DDR cells. 96-well plates were coated with GFOGER or combinations of GMOGER or GAOGER (denoted as GMO and GAO, respectively), GPRGQOGVNleGFO (GVMGFO, denoted as GVM), and GPP, at the indicated ratios. Cells were allowed to adhere for 1 h at 37°C. (A) Cell adhesion to combinations of GMOGER and GVMGFO. (B) Cell adhesion to combinations of GAOGER and GVMGFO. The data are representative of at least four independent experiments, each performed in triplicate. The error bars indicate the sample standard deviation.

**Figure 8:**
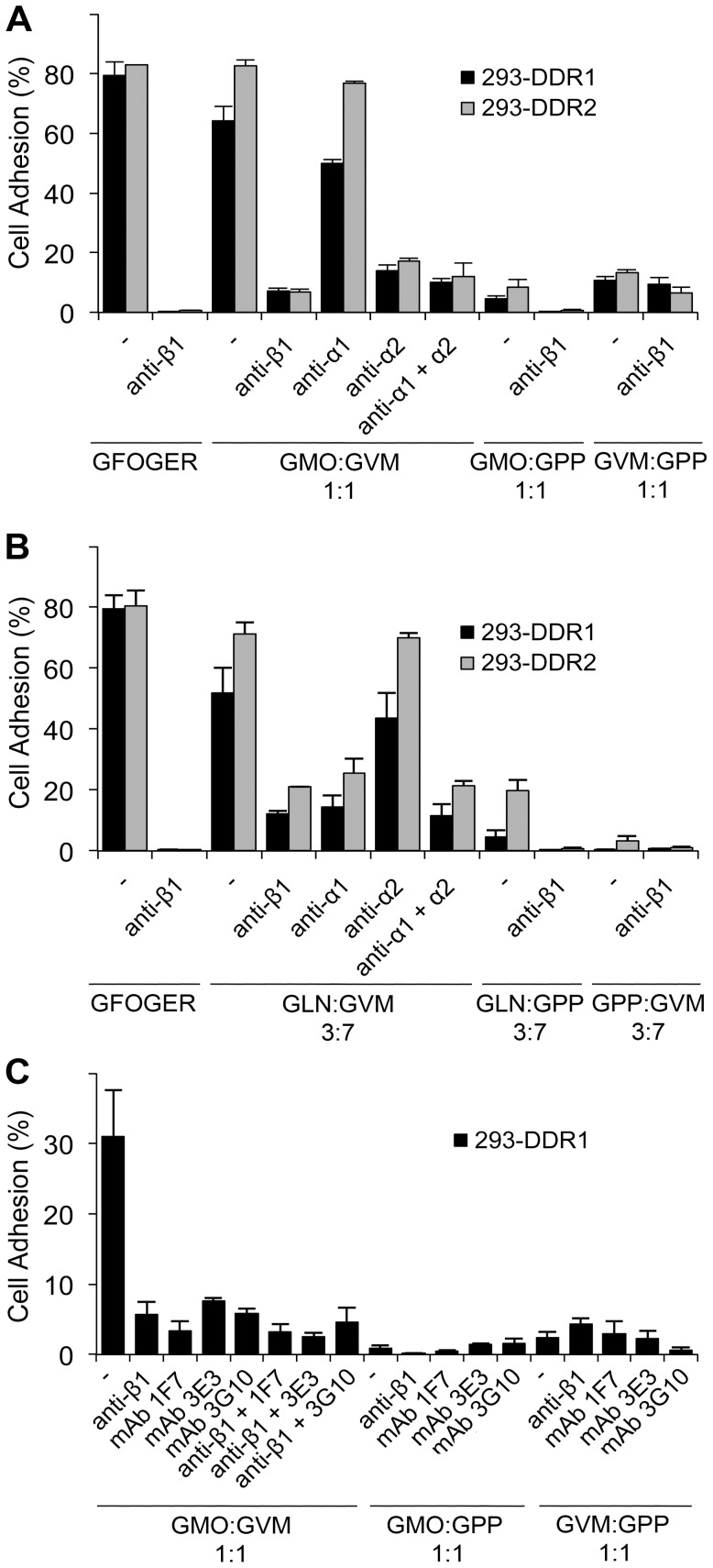
Activation of DDRs enhances integrin-mediated cell adhesion to medium-affinity integrin ligands. 96-well plates were coated with GFOGER or combinations of GMOGER or GLOGEN (denoted as GMO or GLN respectively), GPRGQOGVNleGFO (GVMGFO, denoted as GVM), and GPP at the indicated ratios. Cells were allowed to adhere for 1 h at 37°C in the absence or presence of either 10 µg/ml anti-integrin mAbs or 15 µg/ml anti-DDR1 blocking mAbs as indicated. (A) Blocking of adhesion to combinations of GMOGER and GVMGFO using anti-integrin mAbs. (B) Blocking of adhesion to combinations of GLOGEN and GVMGFO using anti-integrin mAbs. (C) Blocking of adhesion to combinations of GMOGER and GVMGFO with different anti-DDR1 mAbs. Data shown are representative of three independent experiments, each performed in triplicate. The error bars indicate the sample standard deviation.

Triple-helical collagen peptides incorporating GVMGFO motifs not only bind to DDRs with high affinity but are also able to activate the receptors [22], [23] (Fig. S1C, D). We examined whether activating DDRs with such peptides would regulate integrin-mediated cell adhesion. To this end, we coated 96-well plates with mixtures of integrin-specific ligands and the DDR-specific ligand GPRGQOGVNleGFO at different ratios. Integrin and DDR ligands were either mixed together or with the negative control, non-receptor binding peptide GPP, to adjust for different coating concentrations. Cell adhesion to these various substrate mixtures was then assessed. Intriguingly, we found that coating of plates with mixtures of GMOGER with GPRGQOGVNleGFO (at 1∶1 and 1∶4 ratios) led to a dramatic increase in cell adhesion of DDR-transfected cells, compared to their adhesion to either GMOGER or GPRGQOGVNleGFO alone, at the same coating concentrations (Fig. 7A). This effect was specific to DDR-expressing cells, since control cells showed poor adhesion to either substrate, as well as to the mixtures. Similar results were also observed for DDR-expressing cells, adhering to the low affinity integrin ligand GAOGER, mixed with GPRGQOGVNleGFO at 1∶1 ratio (Fig. 7B), or to the α1β1 integrin ligand GLOGEN, mixed with GPRGQOGVNleGFO at 1∶1 ratio (Fig. 8B). Further Ab blocking assays revealed that the enhanced adhesion to the peptide mixtures could be inhibited by anti-β1 blocking Ab (Fig. 8A & B), indicating that the enhanced adhesion is integrin-mediated rather than DDR-mediated. Moreover, in line with the receptor specificity of GMOGER and GLOGEN, anti-α2 Ab, but not anti-α1 Ab, blocked the enhanced adhesion to the GMOGER : GPRGQOGVNleGFO mixture, whereas in the case of the GLOGEN : GPRGQOGVNleGFO mixture, it was anti-α1, not anti-α2, that inhibited the enhanced adhesion.

In an attempt to block DDR receptor signalling, we used a number of anti-DDR1 blocking mAbs that were generated in our lab [15]. These mAbs inhibit DDR1 signalling allosterically, without blocking the DDR1-collagen interaction [15]. As shown in Fig. 8C, the enhanced cell adhesion was substantially reduced after pre-incubating cells with these blocking Abs. Taken together, these data show that activation of the DDRs by GPRGQOGVNleGFO, which induces receptor signalling, enhances α1β1 and α2β1 mediated cell adhesion.

## Discussion

The DDRs are thought to play major roles in cell adhesion in a variety of cell types and several studies have suggested that DDR1 functions as an adhesion receptor (e.g. refs [31], [35], [47]). However, the role of the DDRs in cell adhesion has so far only been studied indirectly and very little is known about their ability to modulate the activity of established adhesion receptors such as integrins. The DDRs and collagen-binding integrins recognize the same ligands, and functional overlap between these two types of receptors has made the analysis of their individual contributions to cell adhesion difficult. A parallel problem in platelets led to the development of the two-site, two-step model used to describe their activation by collagen [48], [49], subsequently found to depend on the signalling receptor, Glycoprotein VI, which in turn activates the adhesion receptor, α2β1. The precise relationship between these receptors is not yet fully defined, and remains the subject of debate, but platelets deficient in each receptor from both human patients [50], [51] or knockout mouse models [52], [53] express defective responses to collagen and bleeding defects of varying degrees of subtlety.

Here, we used synthetic triple-helical collagen-derived peptides that selectively target either DDRs or integrins in order to resolve the input of each receptor to cell adhesion and to analyze a potential synergy between them. Collagen-mimetic peptides of similar design have been shown to effectively capture certain functions of native collagens, for example in mediating platelet adhesion and thrombus formation [11], [54]. Receptor-selective collagen peptides of the same kind as used here allowed independent control over different collagen receptors to define synergism between platelet collagen receptors [55]. Moreover, triple-helical peptides containing high-affinity integrin motifs such as GFOGER are widely used in both basic science studies and tissue engineering applications. The latter peptides are known to possess cell adhesive functions similar to full-length collagen in that cells can firmly attach and spread on these substrates (e.g. refs [56], [57], [58]).

Here we show that in HEK293 cells collagen-binding β1 integrins play a primary role in mediating cell adhesion to collagen I. The DDRs, on the other hand, only mediate modest cell binding to the DDR-selective GVMGFO motif in fibrillar collagens (Fig. 1). While integrin-mediated cell adhesion is sensitive to inhibition by dasatinib, cell attachment to the GVMGFO motif is independent of DDR transmembrane signalling as dasatinib, a potent DDR kinase inhibitor, had no effect on this interaction (Fig. 2). Integrin-mediated cell adhesion involves both ligand binding and cell spreading. Dasatinib blocks Src and focal adhesion kinases, and exerts its effect on cell adhesion by inhibiting events downstream of integrin-mediated ligand binding [44], such as cell spreading. The inability of dasatinib to inhibit cell binding to GPRGQOGVNleGFO, together with our observations that cell attachment to this substrate does not lead to cell spreading (Fig. 3), indicates that the DDRs, unlike integrins, cannot establish proper cell-matrix contacts, which would trigger a signalling cascade that leads to actin polymerisation and myosin contraction, to effect the morphological changes that result in cell spreading. We conclude that, while the DDRs can mediate a passive interaction of cells with collagen, they are not true adhesion receptors.

In this report we demonstrate a positive role for the DDRs in modulating integrin-mediated cell adhesion and cell spreading. As has been observed with GFOGER containing collagen-mimetic peptides (e.g. ref [58]), the GVMGFO-containing peptide could effectively inhibit adhesion of DDR2-expressing cells to collagen I in a competition assay (Fig. 4). While we consistently (in 5 experiments) observed a blocking effect of the GVMGFO peptide on cell adhesion to collagen by the DDR2 expressing cells, the magnitude of inhibition varied between ∼20–80% inhibition. For DDR1 expressing cells, on the other hand, a blocking effect was only observed in 2/4 experiments (data not shown). The reasons for the less robust effect on DDR1 expressing cells are not clear, but it is possible that DDR2 expressing cells express more DDR2 receptors compared with DDR1 receptors on DDR1 expressing cells, which would also help explain the higher adhesion levels of DDR2 expressing cells to low- and intermediate-affinity substrates (e.g. Fig. 5). Our experiments suggest that the DDRs actively participate in cell adhesion to collagen. We further show that overexpression or activation of the DDRs with GPRGQOGVNleGFO leads to increased adhesion to low- and intermediate-affinity integrin ligands, via α1β1- or α2β1-mediated cell adhesion (Figs 5, 7, 8). In contrast to cell attachment to the GVMGFO site, for which DDR signalling is not required, co-activation of the DDRs and integrins with GPRGQOGVNleGFO and GMOGER (or GAOGER/GLOGEN) promotes enhanced α2β1- (or α1β1-) mediated cell adhesion that is dependent on DDR signalling (Fig. 8C).

Here we show that the DDRs can cooperate with the collagen-binding integrins α1β1 and α2β1 by strengthening their adhesion to collagen. Collagen-integrin interactions are defined by both the affinity of the integrin ligand and the activation state of the receptor. Integrins can display different conformations relating to low- and high-affinity states [59], [60]. We used a set of collagen-mimetic peptides with different affinities for α2β1 and α1β1 to probe integrin activation. Binding and adhesion to the high-affinity GFOGER peptide does not require integrin activation, whereas binding to medium- or low-affinity ligands requires prior activation of the receptor [11]. The cellular context defines the activation state of collagen-binding integrins. For example, α2β1 on resting platelets is present in a low activation state, in which it can bind the high-affinity GFOGER but less well or not at all to medium- or low-affinity collagen-like peptides such as GLOGER, GMOGER and GAOGER. Platelet activation enhances adhesion to the latter peptides. On HT1080 cells, on the other hand, α2β1 is found in a more active conformation and is able to adhere to GFOGER, GLOGER and GMOGER to similar extents, without cellular activation [11]. Our data show that both DDRs promote cell adhesion to the medium- or low-affinity integrin ligands GMOGER, GAOGER and GLOGEN (Figs 5, 7 and 8), without enhancing the expression levels of α1β1 or α2β1 (Fig. 6). Since we observe enhanced basal cell adhesion to integrin ligands that requires prior activation of the integrin receptors, we conclude that the DDRs enhance the integrin activation state of α1β1 and α2β1. Overexpression of the DDRs thus likely leads to expression of α1β1 and α2β1 in a more active conformation. Activation of the DDRs with GVMGFO further enhances the affinity of α2β1 and α1β1 to their ligands GMOGER, GAOGER and GLOGEN.

Other studies report the use of integrin activation reporter mAbs to directly visualise activated β1 integrins (e.g. refs [61], [62]). Such mAbs bind to integrins in their high-affinity state and thus are deemed activation specific [63]. However, using two of such reporter mAbs against different epitopes in the β1 subunit, we were unsuccessful in our attempts to demonstrate a difference in activated β1 integrins, either between unstimulated 293-EV and 293-DDR1 or 293-DDR2 cells, or between different stimulation regimes using the same cell line (data not shown). All three cell lines displayed low binding of mAbs 9EG7 or 12G10 by flow cytometry and there was no significant increase when cells were plated onto various collagen or peptide substrates. Furthermore, immunofluorescence staining with 12G10 showed a broad range of fluorescence intensities between individual cells, and quantification of staining showed no significant differences between the cell lines adhering to either poly-L-lysine, collagen I or collagen-like peptides. We were also not able to observe an increase in 12G10 staining when cells were treated with either 0.1 mM Mn^2+^ or 50 nM PMA (phorbol myristate acetate), suggesting that the assay was not sensitive enough to detect higher affinity β1 integrins on DDR-expressing 293 cells. We note earlier findings by others that concluded that cellular activation of α2β1, unlike α4β1 or α5β1, only weakly increases the binding of β1 activation-specific mAbs [64]. While such mAbs may be useful tools to detect differences in the activation state of certain β1 integrins (e.g. α4β1), their use may be limited for collagen-binding integrins.

The roles that DDRs play in cell adhesion are most likely cell-type dependent, as various adhesive roles for DDRs in cell adhesion have been reported. Overexpression of DDR1 was shown to promote cell adhesion to collagen I in leukemic THP-1 cells independently of β1 integrin function [31]. DDR1 was also shown to enhance cell adhesion to collagen I in glioma and pituitary adenoma cells [32], [33]. Consistent with an adhesion-promoting role of DDR1, several studies using DDR1 knockout mice have observed reduced adhesion to collagen. In smooth muscle cells from *Ddr1*−/− mice, lack of DDR1 led to decreased cell adhesion to collagens type I and VIII, while adhesion to fibronectin and vitronectin was unaffected [34]. Similarly, mesangial cells from *Ddr1*−/− mice showed reduced adhesion to collagen I [35], while macrophages from *Ddr*1−/− mice had impaired adhesion on collagen IV [65]. Interestingly, in melanocytes, knockdown of DDR1 was shown to reduce adhesion to the basement membrane collagen IV but not to collagen I [66]. In contrast to the above cellular systems where DDR1 plays a positive role in cell adhesion, DDR1 was shown to play a negative role in MDCK cells, where it inhibits α2β1-mediated cell adhesion to collagen I [36]. Further complicating the picture, several studies failed to detect an effect of DDR1 on cell adhesion to collagen I or IV (e.g. refs [67], [68], [69]). The role that DDR2 plays in cell adhesion is not clear. DDR2 is generally expressed in cells of mesenchymal origin, similar to α10β1 and α11β1 [2], [4], [5]. While we have not addressed the ability of DDR2 to modulate α10β1- or α11β1-mediated cell adhesion, it is conceivable that DDR2 affects α10β1 function on chondrocytes or α11β1 function on fibroblasts. However, in contrast to DDR1, DDR2 seems to play no role in smooth muscle cell adhesion to collagen I, as cells from both *Ddr2+/+* and *Ddr2−/−* mice adhered to collagen I to the same extent [70]. Similarly, skin fibroblasts from *Ddr2*−/− mice showed the same level of adhesion to collagen I as those from *Ddr2*+/− mice [71]. Taken together, the physiological roles that the DDRs play in cell adhesion seem to strongly depend on the cellular context.

Although this study does not address the DDR downstream signalling pathways, our results demonstrate a cooperative interplay between the DDRs and α1β1 and α2β1 integrins. Only limited information exists about the intersection of integrin and DDR signalling pathways. DDR1 has been observed to both cooperate with α2β1 and to suppress its function. Cooperation of DDR1 and α2β1 was required in the regulation of cell scattering in pancreatic cancer cells [40] or the self-renewal of mouse embryonic stem cells [41], whereas in MDCK cells, DDR1 was shown to inhibit several α2β1-mediated cell functions including cell adhesion and migration [36]. An interplay between DDR1 and α1β1 has not been described, but DDR2 and α1β1 were shown to both be involved in MMP-13 expression on chondrocytes [72].

In conclusion, our results show that collagen-binding β1 integrins, rather than the DDRs, are the receptors that directly mediate cell adhesion to collagen in HEK293 cells. The DDRs, while being able to mediate cell attachment in a receptor signalling-independent manner, do not support firm adhesion themselves. However, the DDRs actively participate in cell adhesion to collagen I: blocking the cellular interaction to the DDR binding site in collagen decreases cell adhesion and activation of the DDRs enhances α1β1 and α2β1 integrin-mediated cell adhesion by regulating the activation state rather than the expression level of the integrins. These findings provide additional insights into the roles of the DDRs in cell adhesion and their interplay with collagen-binding integrins. While the mechanism by which the DDRs regulate the activation state of collagen-binding integrins has yet to be elucidated, it is conceivable that the contribution of the DDRs may be more prominent in tissues where high-affinity integrin binding sites are either not exposed on collagen or inaccessible.

## Supporting Information

Figure S1 DDR expression and activation by collagen I and collagen-derived synthetic peptides in DDR-expressing HEK293 cell lines. (A) Western blot analysis of lysates from stably transfected HEK293 cells. Total cell lysates were subjected to SDS-PAGE and immunoblotting with the indicated Abs. β-tubulin was monitored as a loading control. (B) Cell surface expression of DDR1b in DDR-expressing or empty-vector control HEK293 cells. The cells were stained on ice with 10 µg/ml anti-DDR1 mAb 1F7 followed by FITC-conjugated goat anti-mouse IgG and analysis by flow cytometry. Open black histograms, secondary Ab only; grey filled histograms, anti-DDR1. Shown are representative data of three independent experiments. (C) and (D) Collagen I and the DDR-activating collagen-derived peptide GVMGFO induces receptor phosphorylation in the DDR-expressing cell lines. (C) 293-DDR1 cells were stimulated for 90 min with collagen I at 10 µg/ml or collagen peptide at 100 µg/ml, cell lysates were subjected to immunoprecipitation with anti-DDR1 Ab and protein A beads. Eluates were analyzed by SDS-PAGE and Western blotting. The blot was probed with anti-phosphotyrosine mAb 4G10 (upper panel), followed by stripping and reprobing with anti-DDR1 (lower panel). (D). 293-DDR2 cells were stimulated for 90 min with collagen I at 10 µg/ml or collagen peptides at 100 µg/ml, and total cell lysates were resolved on two gels. The corresponding blots were probed with anti-phosphotyrosine mAb 4G10 (upper panel) or anti-DDR2 (lower panel). The positions of molecular weight markers (in kDa) are indicated.
(PDF)

Figure S2 Dasatinib inhibits collagen I-induced DDR autophosphorylation. Full length DDR1b (A) or DDR2 (B) were transiently expressed in HEK293 cells. Cells were stimulated with 10 µg/ml collagen I for 90 min in the absence or presence of dasatinib at the indicated concentrations. Aliquots of cell lysates were analyzed by SDS-PAGE and Western blotting. The blots were probed first with anti-phosphotyrosine mAb 4G10 (A & B, upper panels) and reprobed with anti-DDR1 (A, lower panel), or, for anti-DDR2, on a duplicate blot (B, lower panel).
(PDF)

Figure S3 Blockade of cell adhesion to collagen I or GLOGER using function-blocking anti-integrin mAbs. Cells were allowed to adhere to collagen I (A) or GLOGER (B) for 1 h at 37°C in the presence or absence of the indicated anti-integrin mAbs. The remaining cell adhesion was measured and calculated as described in Materials and Methods. Data shown are representative of three independent experiments, each performed in triplicate. The error bars indicate the sample standard deviation.
(PDF)

Table S1 Sequences of synthetic triple-helical collagen-derived peptides used in this study.
(DOCX)
