# Supplementary material for: Discoidin Domain Receptors Promote α1β1- and α2β1-Integrin Mediated Cell Adhesion to Collagen by Enhancing Integrin Activation
Source: PLoS One. 2012 Dec 20;7(12):e52209. doi: 10.1371/journal.pone.0052209 (PMC3527415; doi:10.1371/journal.pone.0052209)
Supplement: Figure S1 — DDR expression and activation by collagen I and collagen-derived synthetic peptides in DDR-expressing HEK293 cell lines. (A) Western blot analysis of lysates from stably transfected HEK293 cells. Total cell lysates were subjected to SDS-PAGE and immunoblotting with the indicated Abs. β-tubulin was monitored as a loading control. (B) Cell surface expression of DDR1b in DDR-expressing or empty-vector control HEK293 cells. The cells were stained on ice with 10 µg/ml anti-DDR1 mAb 1F7 followed by FITC-conjugated goat anti-mouse IgG and analysis by flow cytometry. Open black histograms, secondary Ab only; grey filled histograms, anti-DDR1. Shown are representative data of three independent experiments. (C) and (D) Collagen I and the DDR-activating collagen-derived peptide GVMGFO induces receptor phosphorylation in the DDR-expressing cell lines. (C) 293-DDR1 cells were stimulated for 90 min with collagen I at 10 µg/ml or collagen peptide at 100 µg/ml, cell lysates were subjected to immunoprecipitation with anti-DDR1 Ab and protein A beads. Eluates were analyzed by SDS-PAGE and Western blotting. The blot was probed with anti-phosphotyrosine mAb 4G10 (upper panel), followed by stripping and reprobing with anti-DDR1 (lower panel). (D). 293-DDR2 cells were stimulated for 90 min with collagen I at 10 µg/ml or collagen peptides at 100 µg/ml, and total cell lysates were resolved on two gels. The corresponding blots were probed with anti-phosphotyrosine mAb 4G10 (upper panel) or anti-DDR2 (lower panel). The positions of molecular weight markers (in kDa) are indicated. (PDF) [file pone.0052209.s001.pdf]

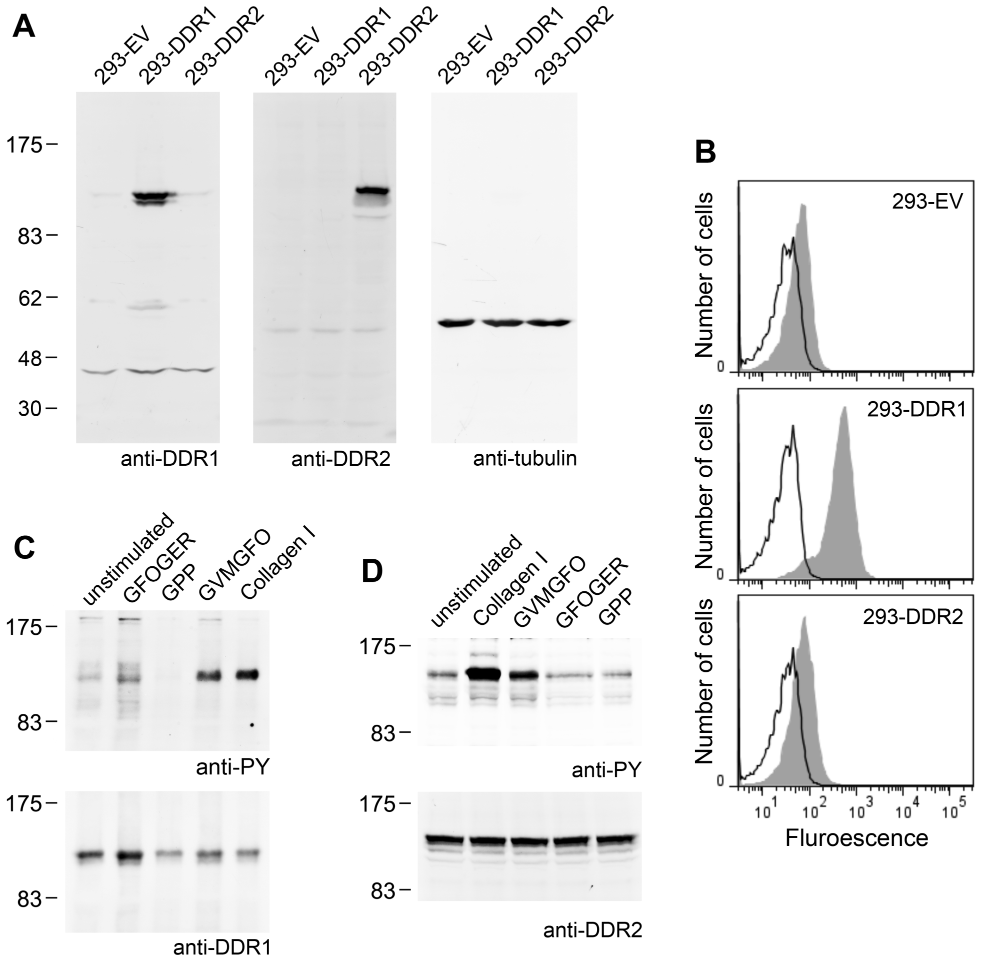

Figure S1. DDR expression and activation by collagen I and collagen-derived synthetic peptides in DDR-expressing HEK293 cell lines. (A) Western blot analysis of lysates from stably transfected HEK293 cells. Total cell lysates were subjected to SDS-PAGE and immunoblotting with the indicated Abs. β-tubulin was monitored as a loading control. (B) Cell surface expression of DDR1b in DDR-expressing or empty-vector control HEK293 cells. The cells were stained on ice with 10 µg/ml anti-DDR1 mAb 1F7 followed by FITC-conjugated goat anti-mouse IgG and analysis by flow cytometry. Open black histograms, secondary Ab only; grey filled histograms, anti-DDR1. Shown are representative data of three independent experiments. (C) and (D) Collagen I and the DDR-activating collagen-derived peptide GVMGFO induces receptor phosphorylation in the DDR-expressing cell lines. (C) 293-DDR1 cells were stimulated for 90 min with collagen I at 10 µg/ml or collagen peptide at 100 µg/ml, cell lysates were subjected to immunoprecipitation with anti-DDR1 Ab and protein A beads. Eluates were analyzed by SDS-PAGE and Western blotting. The blot was probed with anti-phosphotyrosine mAb 4G10 (upper panel), followed by stripping and reprobing with anti-DDR1 (lower panel). (D). 293-DDR2 cells were stimulated for 90 min with collagen I at 10 µg/ml or collagen peptides at 100 µg/ml, and total cell lysates were resolved on two gels. The corresponding blots were probed with anti-phosphotyrosine mAb 4G10 (upper panel) or anti-DDR2 (lower panel). The positions of molecular weight markers (in kDa) are indicated.
