# Supplementary material for: Discoidin Domain Receptors Promote α1β1- and α2β1-Integrin Mediated Cell Adhesion to Collagen by Enhancing Integrin Activation
Source: PLoS One. 2012 Dec 20;7(12):e52209. doi: 10.1371/journal.pone.0052209 (PMC3527415; doi:10.1371/journal.pone.0052209)
Supplement: Figure S2 — Dasatinib inhibits collagen I-induced DDR autophosphorylation. Full length DDR1b (A) or DDR2 (B) were transiently expressed in HEK293 cells. Cells were stimulated with 10 µg/ml collagen I for 90 min in the absence or presence of dasatinib at the indicated concentrations. Aliquots of cell lysates were analyzed by SDS-PAGE and Western blotting. The blots were probed first with anti-phosphotyrosine mAb 4G10 (A & B, upper panels) and reprobed with anti-DDR1 (A, lower panel), or, for anti-DDR2, on a duplicate blot (B, lower panel). (PDF) [file pone.0052209.s002.pdf]

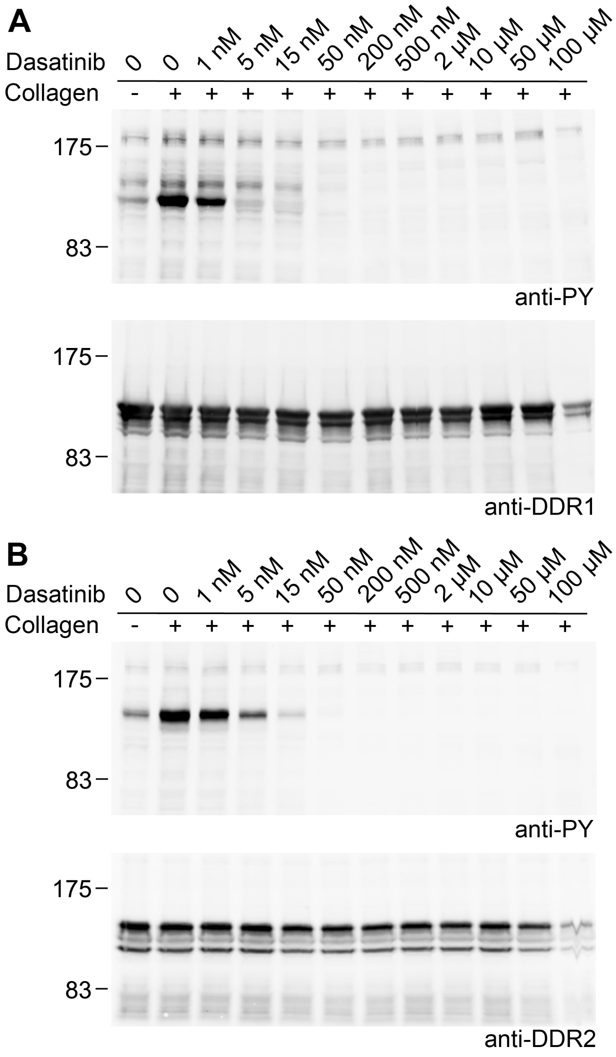

Figure S2. **Dasatinib inhibits collagen I-induced DDR autophosphorylation.** Full length DDR1b (A) or DDR2 (B) were transiently expressed in HEK293 cells. Cells were stimulated with 10 µg/ml collagen I for 90 min in the absence or presence of dasatinib at the indicated concentrations. Aliquots of cell lysates were analyzed by SDS-PAGE and Western blotting. The blots were probed first with anti-phosphotyrosine mAb 4G10 (A & B, upper panels) and reprobed with anti-DDR1 (A, lower panel), or, for anti-DDR2, on a duplicate blot (B, lower panel).
