# Supplementary material for: Discoidin Domain Receptors Promote α1β1- and α2β1-Integrin Mediated Cell Adhesion to Collagen by Enhancing Integrin Activation
Source: PLoS One. 2012 Dec 20;7(12):e52209. doi: 10.1371/journal.pone.0052209 (PMC3527415; doi:10.1371/journal.pone.0052209)
Supplement: Figure S3 — Blockade of cell adhesion to collagen I or GLOGER using function-blocking anti-integrin mAbs. Cells were allowed to adhere to collagen I (A) or GLOGER (B) for 1 h at 37°C in the presence or absence of the indicated anti-integrin mAbs. The remaining cell adhesion was measured and calculated as described in Materials and Methods. Data shown are representative of three independent experiments, each performed in triplicate. The error bars indicate the sample standard deviation. (PDF) [file pone.0052209.s003.pdf]

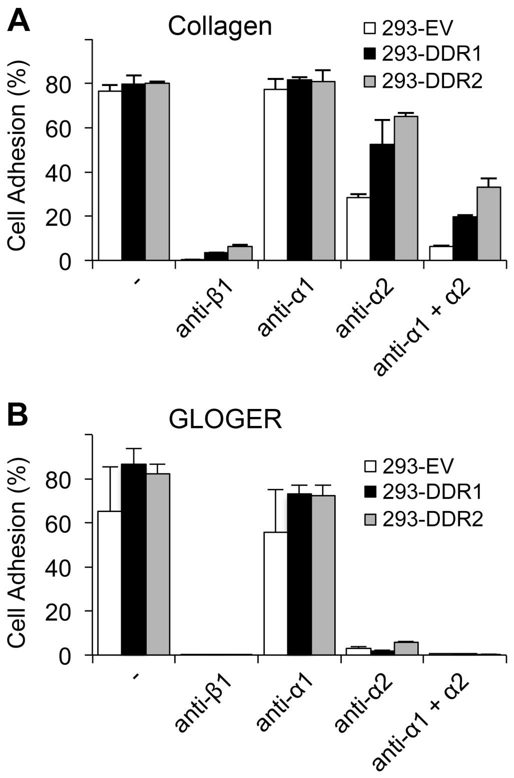

Figure S3. **Blockade of cell adhesion to collagen I or GLOGER using function-blocking anti-integrin mAbs.** Cells were allowed to adhere to collagen I (A) or GLOGER (B) for 1 h at 37 °C in the presence or absence of the indicated anti-integrin mAbs. The remaining cell adhesion was measured and calculated as described in Materials and Methods. Data shown are representative of three independent experiments, each performed in triplicate. The error bars indicate the sample standard deviation.
