# Supplementary material for: Discoidin Domain Receptors Promote α1β1- and α2β1-Integrin Mediated Cell Adhesion to Collagen by Enhancing Integrin Activation
Source: PLoS One. 2012 Dec 20;7(12):e52209. doi: 10.1371/journal.pone.0052209 (PMC3527415; doi:10.1371/journal.pone.0052209)
Supplement: Table S1 — Sequences of synthetic triple-helical collagen-derived peptides used in this study. (DOCX) [file pone.0052209.s004.docx]

**Supplemental Table 1**

**Sequences of synthetic triple-helical collagen-derived peptides used in this study**

| **Name** | **Sequence** |
| --- | --- |
|  |  |
| GVMGFO^[[1]](#footnote-1)^ | GPC(GPP)_5_ -GPRGQOGVNleGFO-(GPP)_5_GPC-NH_2_ |
| GFOGER | GPC(GPP)_5_ -GFOGER-(GPP)_5_GPC-NH_2_ |
| GLOGER | GPC(GPP)_5_ -GLOGER-(GPP)_5_GPC-NH_2_ |
| GMOGER | GPC(GPP)_5_ -GMOGER-(GPP)_5_GPC-NH_2_ |
| GAOGER | GPC(GPP)_5_ -GAOGER-(GPP)_5_GPC-NH_2_ |
| GLOGEN | GPC(GPP)_5_ -GLOGEN-(GPP)_5_GPC-NH_2_ |
| GPP | GPC(GPP)_10_-GPC-NH_2_ |
|  |  |
|  |  |

1. The methionine in GVMGFO was substituted by the isosteric amino acid norleucine (Nle), which increased DDR binding (ref 14). The peptide contained the sequence GPRGQO because of its requirement for DDR activation in cells (ref 22). [↑](#footnote-ref-1)
